# Effect of increasing workload on knee extensor and flexor muscular activity during cycling as measured with intramuscular electromyography

**DOI:** 10.1371/journal.pone.0201014

**Published:** 2018-08-02

**Authors:** Julio Cézar Lima da Silva, Maria M. Ekblom, Olga Tarassova, Eva Andersson, Gustaf Rönquist, Helene Grundström, Anton Arndt

**Affiliations:** 1 The Swedish School of Sport and Health Sciences, GIH, Stockholm, Sweden; 2 School of Physical Education, Physiotherapy and Dance of the Federal University of Rio Grande do Sul, Porto Alegre, Brazil; 3 Department of Neuroscience, Karolinska Institute, Stockholm, Sweden; 4 Department of Radiology, Danderyds Hospital, Stockholm, Sweden; 5 Department of CLINTEC, Karolinska Institute, Stockholm, Sweden; Universita degli Studi di Roma 'Foro Italico', ITALY

## Abstract

The purpose of this study was to describe the effect of increasing workload on individual thigh muscle activation during a 20 minute incremental cycling test. Intramuscular electromyographic signals were recorded from the knee extensors rectus femoris, vastus lateralis, vastus medialis and vastus intermedius and the knee flexors semimembranosus, semitendinosus, and the short and long heads of the biceps femoris during increasing workloads. Mean activation levels were compared over the whole pedaling cycle and the crank angles at which onset and offset of activation and peak activity occurred were identified for each muscle. These data were compared between three workloads. EMG activation level significantly increased (p<0.05) with increasing workload in the rectus femoris, vastus medialis, vastus lateralis, vastus intermedius, biceps femoris long head, semitendinosus and semimembranosus but not in the biceps femoris short head. A significant change in activation timing was found for the rectus femoris, vastus lateralis, vastus medialis and semitendinosus. Of the knee flexors only the short head of the biceps femoris had its peak activity during the upstroke phase at the two highest workloads indicating a unique contribution to knee flexion.

## Introduction

In cycling, muscle recruitment behavior and muscle coordination have been shown to depend upon training level, cycling modality and intensity [1]. Changing the resistance imposed while pedaling is an effective strategy for changing the level of muscle activity [2, 3].

In training and racing, muscle activation will need to adapt to changes in workload and muscular fatigue [4]. A number of studies have shown an increase in muscle activation amplitude as measured via surface electromyography (EMG) when increasing the workload [3, 5–12]. With increasing fatigue the cyclist requires a neuromuscular strategy for modifying motor unit recruitment and consequently EMG activity to maintain the greatest possible power production [13]. Such adaptations might have consequences upon the synergy between deep and superficial muscles and there is at present little research on the timing of superficial and deep thigh muscle activation during cycling [13].

The activity of the deep muscles vastus intermedius (Vint) and the short head of the biceps femoris (BFS) has recently been investigated using intramuscular electromyography during cycling at a single workload (190 W) [14]. The Vint was predominantly activated during the initial part of the downstroke phase together with vastus lateralis (VL), vastus medialis (VM) and rectus femoris (RF). The BFS was activated somewhat later in the crank cycle and not always in synergy with the biceps femoris long head (BFL), semimembranosus (SemM) and semitendinosus (SemT) [14]. However, information on how these synergies are affected by increasing workload and the related greater perceived exertion has not previously been published.

Intramuscular EMG significantly reduces the risk for contaminated signals from adjacent muscles, which may occur when using superficial EMG [15]. A further advantage of intramuscular EMG is the possibility to evaluate deep muscles with difficult access for superficial EMG electrodes. Conversely, fine-wire electrodes only register activity in a small volume of the muscle, while superficial EMG can cover a larger surface area of a muscle representing a more global activation behavior of the specific muscle. In order to investigate the muscular coordination of mono- and bi-articular, deep and superficial muscles during pedaling at increasing workloads, the present study used intramuscular EMG with fine-wire electrodes inserted directly in the relevant muscles.

The purpose of this study was to describe the effect of increasing workload on individual thigh muscle activation during a 20 minute incremental cycling test.

Based on a recent investigation of cycling at a submaximal workload, which showed that the Vint and BFS muscles are generally, but not always, active in synergy with the superficial knee extensors and flexors, respectively [14], we hypothesized that the deep Vint muscle would be activated in synergy with the superficial knee extensors and the BFS in synergy with the superficial knee flexors across all workloads. Furthermore, based on the results of previous studies using surface EMG, we hypothesized that increasing workload would result in increased activation of the deep and superficial knee extensor and flexor muscles, without alterations in the crank angle at which activation onset or offset or peak activation occurred.

## Materials and methods

The present study was part of a series of studies investigating neural activation of the thigh muscles using intramuscular EMG. This series also included a previously published study on neuromuscular coordination during cycling at 190 W [14] and studies on dynamic and isometric rehabilitation exercises.

### Participants

Nine male athletes active in recreational cycling or triathlon were asked to participate in the study (mean ± SD): age 31.7 ± 10.9 years, body mass 79.7 ± 4.7 kg, height 182.7 ± 7.4 cm). The sample size was similar to that investigated in previous intramuscular activation studies reporting the reliability of this technique [16, 17]. Sample sizes in these studies are often restricted due to the invasive nature of the experimental procedure. Participants provided their written informed consent and the study was approved by the Stockholm regional ethical committee (approval nr: 2014/641-31/1). Participants chose their individual preferred sitting position on the ergometer.

### Protocol

A 10 minute warm-up on a bicycle ergometer (LC4, Monark Exercise AB, Sweden) was followed by the incremental cycling test with a maximum duration of 20 minutes. The first workload of the cycling protocol was 170 W with 20 W increments every two minutes automatically controlled by Monark 939E analysis software (Monark Exercise AB, Sweden). The maximum workload at 20 min was therefore 350 W. Seven participants completed the complete protocol, while two participants experienced fatigue before the 20 min maximum time and could not cycle at the highest workloads (the maximum workloads for these two participants were 310 W and 270 W). Cycling shoes with SPD cleats were used by all participants. A pedaling cadence of 90 rpm was maintained throughout the incremental test and controlled using visual feedback from a monitor on the handlebars. Participants indicated their perceived effort on the Borg scale at each workload increase [18]. The data presented in this study were from the 190 W (defined as initial) and final (270–350 W) workloads. The workload midway between the initial and individually achieved final workloads was chosen as the intermediate workload (230–270 W).

### Data collection

Heart rate (bpm) was registered by the Monark 939E analysis software (Monark Exercise AB, Sweden) using a chest belt (Premium heart rate monitor, Garmin, USA). Intramuscular EMG (MyoSystem 1400A, Noraxon Inc., USA) was used to record the muscle activation. The intramuscular EMG technique has been described in detail in a previous study [14]. In brief, two teflon coated silver wire electrodes were placed into each muscle under ultrasound guidance with a final inter tip distance of 5–10 mm. A non-optimal electrode location for the first five participants was observed in the BFS, so these data were excluded from further analysis. EMG signals were recorded at 5000 Hz per channel using a 16 bit, analog to digital converter (Power 1401, Cambridge Electronic Design (CED), England) in Spike2 software (v7.0, CED, England). EMG data were recorded during the last 20s of the initial, intermediate and final workloads of the incremental test.

Right lower limb and pedal cycle kinematics were acquired using five optoelectronic cameras (Oqus 4-series, Qualisys AB, Sweden), recording at 250 frames per second via the Qualisys Track Manager (QTM^®^) software (Qualisys AB, Sweden). Spherical reflective markers (12 mm diameter) were placed on the acromion, anterior iliac spine, greater trochanter, thigh, lateral femoral condyle, lateral malleolus, crank axis and pedal. The kinematic data were recorded during the same 20 s as the EMG data collection and an analog signal was used to synchronize all data in the QTM software. The global sagittal plane was defined by the plane formed by the rotation of the right pedal defined by the markers on the crank axis and pedal to correct for any deviation of the bicycle orientation relative to the calibrated global coordinate system. The crank angles were defined as the position of the crank in the pedal cycle in this adjusted plane with 0° (360°) representing top dead center (TDC). The crank angle was used to divide the data into single, consecutive crank revolutions.

### Data analysis

A band-pass Butterworth filter (50–1000 Hz) and a root mean square (RMS) envelope with a window of 20 ms were applied to the EMG signals. The RMS data of each muscle were normalized to the highest peak of the average RMS curve of ten consecutive crank revolutions registered in any of the three workloads [19, 20]. The mean normalized EMG RMS over the whole crank cycle was then calculated and averaged over the 10 crank revolutions. This normalized EMG RMS over the whole cycle is from now on referred to as the mean EMG activation level. The crank angles for peak activity were calculated from the normalized RMS data of each muscle for every participant prior to averaging across the ten crank cycles and all participants.

The definition of the times for EMG onset (ON) and offset (OFF) has been described in previous studies [14, 21]. In brief the thresholds for ON and OFF were defined individually as 20% of the maximum amplitude found at any workload. A burst was defined when the signal was below 10% and increased to over 20% of this maximum amplitude and remained at this level for longer than 10% of the crank cycle. If the signal decreased below 10% of the maximum amplitude but within 1% of the crank cycle duration rose above 20% maximum amplitude again this was defined as a continued burst. EMG data analysis was conducted using Spike2 (CED, England) and Matlab (Mathworks, USA) software.

The marker positions for the kinematic data were automatically tracked in the QTM software. A digital second-order zero lag low-pass Butterworth filter with cut-off frequency of 10 Hz was used to smooth the kinematic data in the Visual 3D software (C-Motion Inc, USA). Hip and knee joint angles were calculated in the corrected sagittal plane defined by the pedal rotation. Full hip extension was defined as 180° and full knee extension as 0°. The average for ten consecutive crank revolutions for each subject was calculated.

### Statistical analysis

Normal distribution of the data and data sphericity were verified by the Shapiro-Wilk and Mauchly tests respectively. Greenhouse-Geisser correction was used when the data did not show sphericity. Outliers in the mean EMG activation level were removed if they deviated from the mean by more than two standard deviations. Statistical significance of differences in heart rate, perceived effort, mean EMG activation level, angle for peak activation and onset and offset of individual muscles between different workloads were determined by separate repeated measure ANOVA’s (for the factor workload) for each dependent variable. When a main effect of workload was identified, differences between workloads were tested using a post hoc Bonferroni test. Significant differences were assumed when p≤0.05. Statistical analysis was conducted in SPSS for Windows (version 21.0, SPSS, IBM Inc, USA) and data are presented as means and standard deviations.

## Results

Heart rate and perceived effort increased significantly with increasing workload (Table 1).

**Table 1:** Heart rate and perceived effort (mean ±SD).

	Initial Workload	Intermediate Workload	Final Workload	p values
**Heart rate (bpm)**	114.3 ± 44	139.8 ± 53.3	160.8 ± 61.3	*, # < 0.001 £ = 0.001
**Perceived effort**	10.6 ±2.2	14.3 ±1.5	19 ±1.3	* = 0.001 #, £ < 0.001

The perceived effort was measured using the Borg scale.

* = Significant difference between the initial and intermediate workloads.

# = Significant difference between the initial and final workloads.

£ = Significant difference between the intermediate and final workloads.

**Fig 1:**
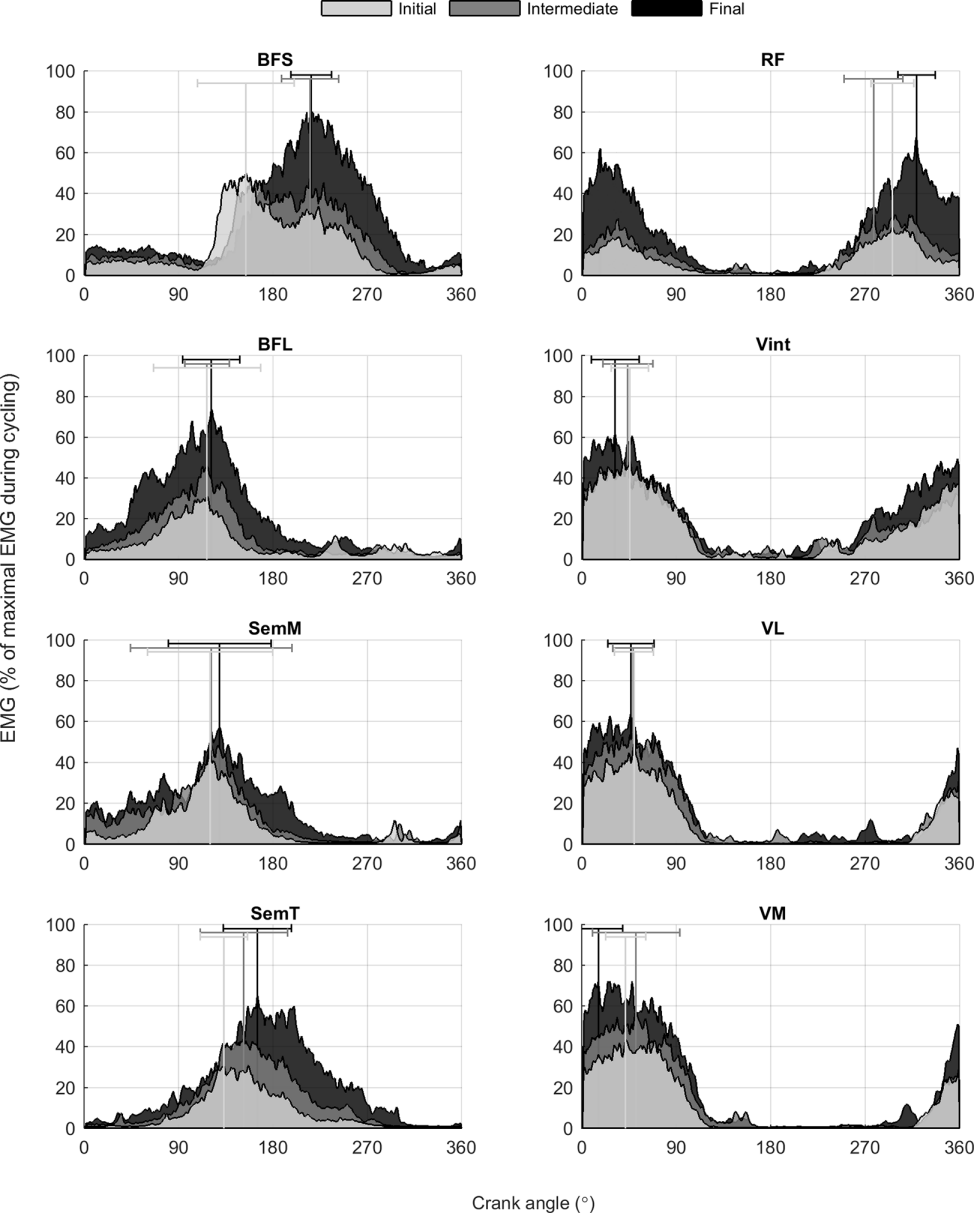
Muscle activation at initial, intermediate and final workloads during the pedaling cycle. Normalized mean activity for biceps femoris short head (BSF), biceps femoris long head (BFL), semimembranosus (SemM), semitendinosus (SemT), rectus femoris (RF), vastus intermedius (Vint), vastus lateralis (VL) and vastus medialis (VM) for all participants. Vertical lines represent the crank angle of peak activity from the mean curve across all participants.

The individual thigh muscles’ activity for all participants adapted differently to increasing workload during different phases of the pedaling cycle (Fig 1).

### Mean EMG activation level

All superficial muscles showed significant adaptations with increased workload, while for the deep muscles significant changes in mean EMG activation were only found for the Vint.

**Fig 2:**
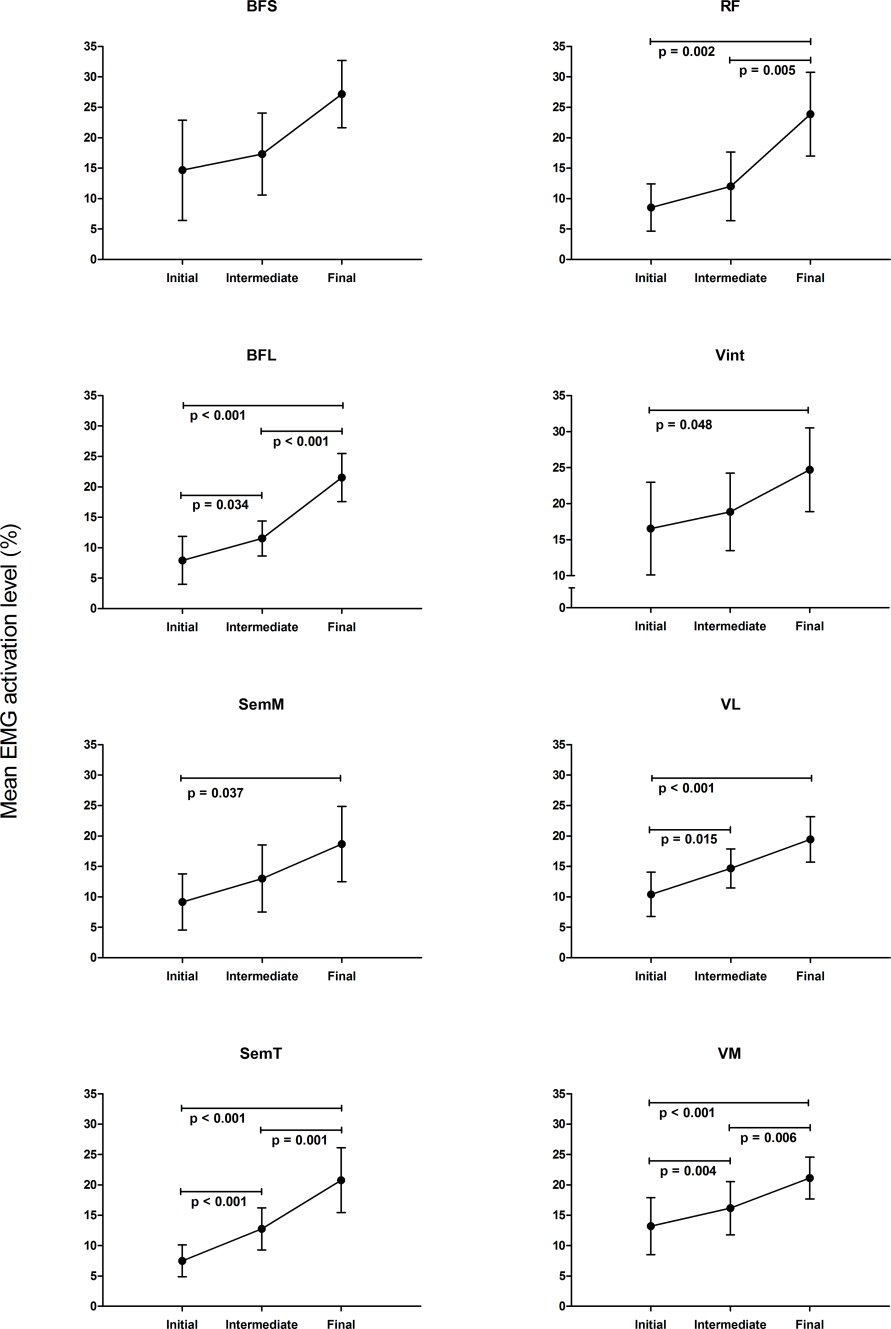
Mean EMG activation level (mean ± SD) at initial, intermediate and final workloads. Normalized mean activation level for biceps femoris short head (BFS), biceps femoris long head (BFL), semimembranosus (SemM), semitendinosus (SemT), rectus femoris (RF), vastus intermedius (Vint), vastus lateralis (VL) and vastus medialis (VM) for the complete pedaling cycle. Horizontal lines indicate statistically significant differences between workloads (p<0.05).

Among the superficial knee extensor muscles, the mean EMG activation level increased significantly with increasing workload for RF (initial = 8.54 ± 3.88, intermediate = 12.02 ± 5.64 and final = 23.88 ± 6.89), VL (initial = 10.42 ± 3.65, intermediate = 14.67 ± 3.20 and final = 19.44 ± 3.73) and VM (initial = 13.20 ± 4.70, intermediate = 16.15 ± 4.39 and final = 21.12 ± 3.46) (Fig 2 and S1 Table).

Among the superficial knee flexor muscles, mean EMG activation level increased significantly with increasing workload for BFL (initial = 7.92 ± 3.93, intermediate = 11.52 ± 2.89 and final = 21.53 ± 3.97), SemT (initial = 7.48 ± 2.63, intermediate = 12.75 ± 3.46 and final = 20.76 ± 5.35) and SemM (initial = 9.17 ± 4.62, intermediate = 13.01 ± 5.51 and final = 18.68 ± 6.19) (Fig 2 and S1 Table).

The mean EMG activation of the deep BFS (initial = 14.67 ± 8.23, intermediate = 17.32 ± 6.73 and final = 27.17 ± 5.53) was not statistically affected by increasing workload, while the mean Vint EMG activation (initial = 16.54 ± 6.43, intermediate = 18.86 ± 5.39 and final = 24.70 ± 5.80) increased at increasing workload (Fig 2 and S1 Table).

### EMG activation timing

There was some inter-individual variation in the EMG activation timing, with some individuals showing a double activation pattern or a lack of activation burst at some workloads (S1 Fig). The statistics underlying the results presented below are performed on the individuals showing a single burst pattern for all three workloads.

**Fig 3:**
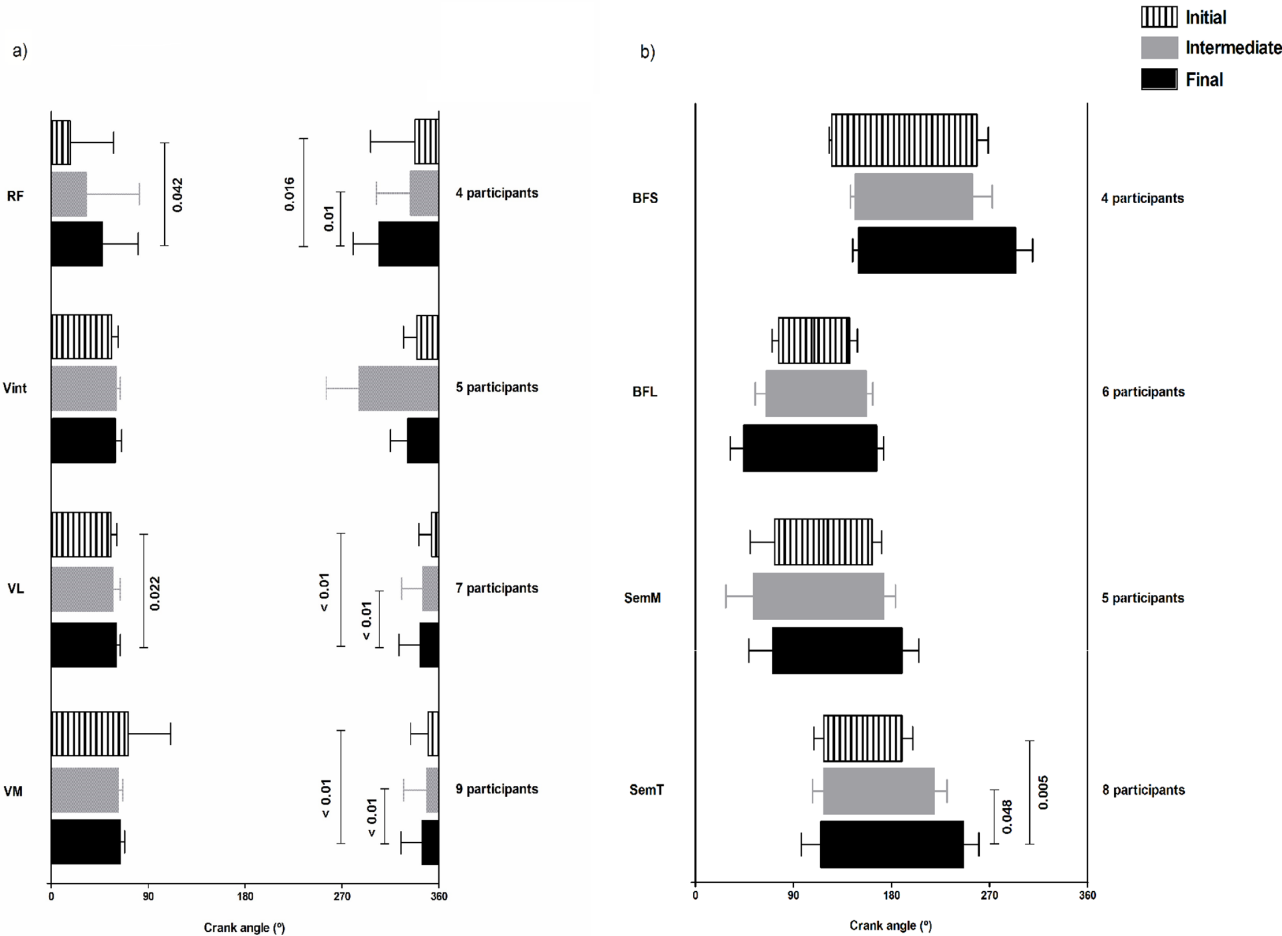
Activation onset and offset (mean ± SD) at initial, intermediate and final workloads. EMG activation onset and offset for (a) knee extensors and (b) knee flexors during the pedaling cycle. Vertical lines indicate statistically significant differences between workloads (p<0.05).

Among the knee extensors, there was a significant main effect of workload on the onset and offset for the RF and VL, and only on the onset for the VM (Fig 3A and S2 Table).

The four participants that showed a single burst across all workloads for RF, displayed a significant difference in the crank angle for onset between the initial (319° ± 75°) and final (259° ± 44°) workloads, and between the intermediate (312° ± 58°) and final workloads. The crank angle for offset was significantly different between the initial (31° ± 74°) and final (85° ± 61°) workloads.

The seven participants that showed a single burst across all workloads for VL, displayed a significant difference in the crank angle for onset between the initial (348° ± 22°) and final (328° ± 36°), and between the intermediate (333° ± 36°) and final workloads. The crank angle for offset was significantly different between the initial (100° ± 10°) and final (109° ± 7°) workloads.

The nine participants showing a single burst across all workloads for VM, only displayed a significant difference in the crank angle of onset between the initial (342° ± 30°) and final (332° ± 36°) workloads, and between the intermediate (340° ± 40°) and final workloads.

Among the knee flexors, only the SemT showed a main effect of workload on activation timing (Fig 3B and S2 Table).

For the eight participants showing a single burst of SemT activation, a significant difference in the crank angle for offset was found between the initial (117° ± 18°) and final (114° ± 36°) workloads, and between the intermediate (117° ± 21°) and final workloads.

As illustrated in Fig 3A and S2 Table the deep Vint was activated in synergy with superficial knee extensors at all workloads. In contrast Fig 3B and S2 Table indicates considerably less synergy between the deep BFS and the superficial knee flexors at all workloads.

**Fig 4:**
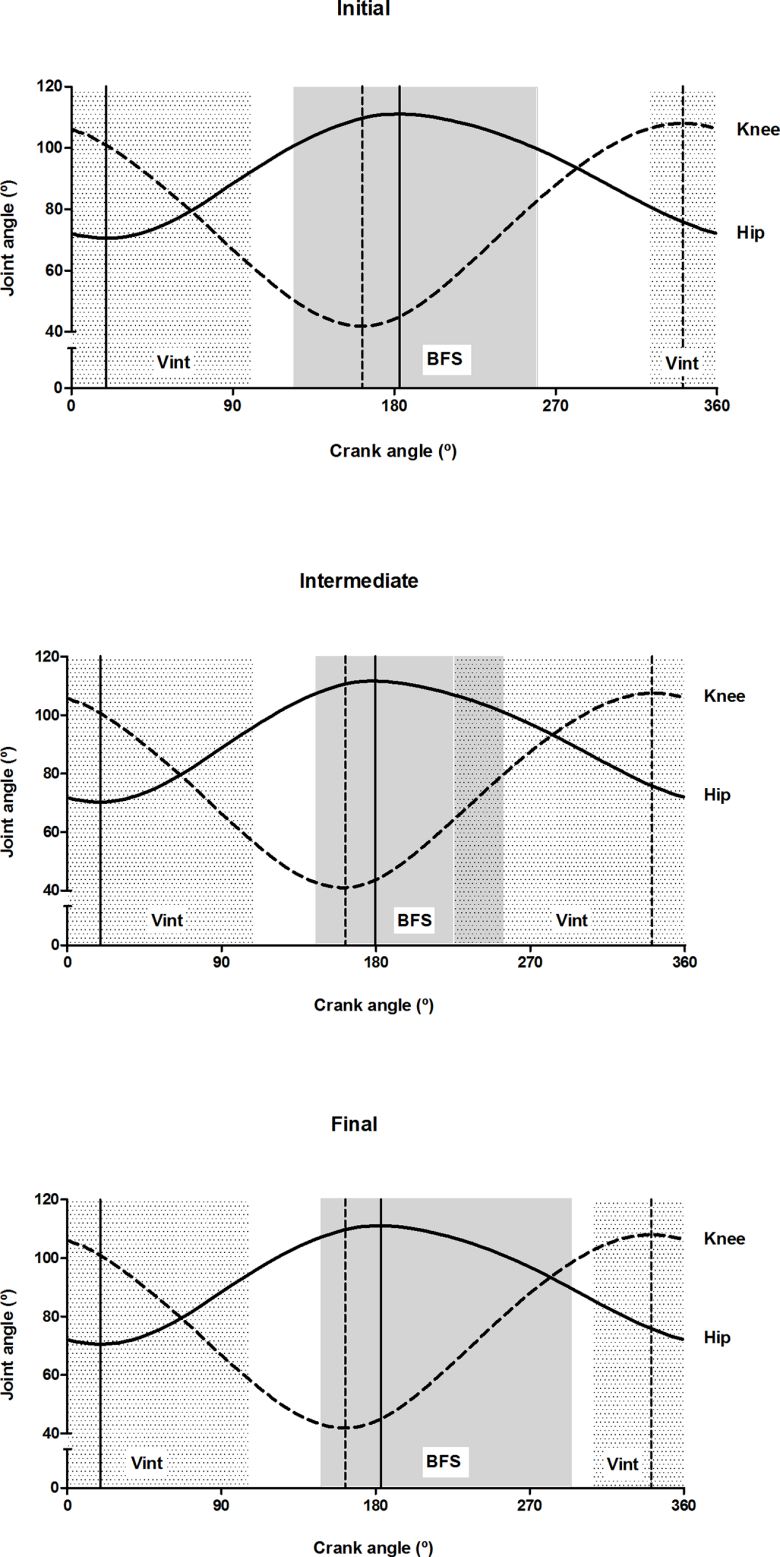
Hip and knee joints angles at initial, intermediate and final workloads. Periods of activation for deep muscles (Vint and BFS) are indicated by the shaded areas and vertical lines represent the crank angle for peak extension and flexion for the hip (solid line) and knee (dotted line) joints.

Fig 4 illustrates the activity bursts of the deep muscles (Vint and BFS) in relation to the hip and knee joint angles. The Vint was active during the transition from knee flexion to extension and hip flexion to extension at all workloads. The BFS was active predominantly around the bottom dead center at the initial workload, corresponding to the transition from knee extension to flexion and hip extension to flexion. The BFS continued its activation during knee flexion at crank angles at which the other knee flexors had ceased to be active (cf. also Figs 1 and 3 and S2 Table).

### Crank angle for peak activity

The crank angle at which peak SemT activity occurred differed significantly with increasing workload (Table 2 and S3 Table). The crank angles at which peak muscle activation occurred did not differ with workload for any other muscle.

**Table 2:** Crank angles at which mean peak activity (mean ± SD) occurred.

	Initial workload	Intermediate workload	Final workload	p values
Muscles	Crank angle for mean peak activity (º)	Crank angle for mean peak activity (º)	Crank angle for mean peak activity (º)	
**Knee flexor**				
BFS	123 ± 46	198 ± 27	224 ± 19	
BFL	111 ± 51	105 ± 21	108 ± 27	
SemM	139 ± 59	124 ± 77	104 ± 49	
SemT	132 ± 22 #	164 ± 42	159 ± 32 #	# 0.019
**Knee extensor**				
RF	323 ± 56	330 ± 64	335 ± 42	
Vint	343 ± 70	8 ± 60	22 ± 34	
VL	32 ± 25	43 ± 19	12 ± 43	
VM	47 ± 19	61 ± 42	35 ± 39	

BFS (biceps femoris short head); BFL (biceps femoris long head); SemM (semimembranosus); SemT (semitendinosus); RF (rectus femoris); Vint (vastus intermedius); VL (vastus lateralis); VM (vastus medialis).

# = significant difference between initial and final workloads.

## Discussion

The main finding of this study was that the Vint was activated in synergy with the superficial knee extensors, whereas the synergy between BFS and superficial knee flexors was considerably less. The mean EMG activation level of the deep BFS was not affected by increasing workload. However the deep Vint was affected by increasing workload. The superficial RF, VL, VM, BFL and SemT and SemM muscles all increased their mean EMG activation level with increasing workload. Care needs to be taken in the interpretation of results due to the low sample size.

There was substantial inter individual variability in the onsets and offsets of the individual muscles, with some displaying two activation burst, while others displayed a single. This presumably signifies that variations between individuals in muscle properties and other anthropometric characteristics might affect the strategy used by the nervous system to perform the same task. Among the individuals showing a single burst at all workloads, the mono-articular knee extensors (VL and VM) showed a statistically significant difference in activation timing together with the bi-articular RF at increasing workloads, without changes in the crank angle of peak activity. SemT was the only knee flexor showing a significant difference in activation timing and was the only muscle with differences in the crank angle of peak activity at increasing workload.

A number of studies have reported increased knee extensor activity at increasing cycling workloads [3, 22–24]. Ericson, Nisell [3] observed a significant increase in activation at 240 W compared to 120 W for the RF and VM. The present study showed that muscle activation also increases at considerably higher intensities (range: 190–350 W). The VL and VM muscles have previously been shown to increase their activation level with increasing intensity [4, 25, 26]. In the present study the VM showed an increase of 8% mean EMG activation level from initial to final workload, whereas the mean VL EMG activation increased 9% at the corresponding workloads.

The bi-articular RF increased its mean EMG activation level across workloads and its activation pattern was similar to the results reported by Blake, Champoux [9] who suggested that the RF contribution to the power output during cycling increases already before the TDC and continues into the downstroke phase. Similarly Blake, Champoux [9], Candotti, Loss [27] also suggested that the greatest contributions of the VL and VM to power output occurred during the downstroke phase, corresponding to the highest activation reported here.

At higher workloads the activation timing for knee extensors has been shown to shift to earlier positions in the crank cycle [25, 26, 28]. This shift to an earlier onset was seen among the individuals displaying a single EMG activation burst for RF, VL and VM with a delayed offset also found for RF and VL from initial to final workload.

The deep Vint was activated in close synergy with RF before the TDC and with VL and VM during the downstroke phase at all workloads. This activation of the Vint before the TDC may be due to a deep knee stabilizing role during a pedal phase where the dominant thigh muscle action is hip flexion by the RF and where knee extension does not appear to be required for torque production, which might indicate a non direction specific stabilizing function of the Vint. This may be a similar local stabilizing role that the deep muscles are assumed to have in cervical and lumbar spine regions, compared to the more superficial direction specific muscles [29, 30].

Both RF and Vint showed a crank angle for peak activity before the TDC at the initial workload while at the final workload the peak activity of the Vint tended to occur during the propulsive phase together with the vastii muscles. The results presented here suggest that at higher intensity the Vint muscle tends towards a modification in muscle coordination to maintain power output. This appears to be more in synergy with the vastii muscles at higher workloads. The longer RF activation timing at higher workloads may indicate an increased contribution to hip flexion and may also assist in coordination of the lower limb prior to the propulsive phase. A previous study reported a double EMG burst in three participants and a single EMG burst in the six participants for RF during a lower workload (190 W) [14]. However, in the present study a double burst was seen not only for the RF, but also for other muscles. This discrepancy can be explained by the different maximum EMG values being used in the two studies for normalization and calculation of the thresholds defining onset and offset. The statistical analysis was conducted only for participants that showed single bursts for all workloads.

The BFL, SemM and SemT also demonstrated significant increases in mean EMG activation level with increasing workload, which is in accordance with previous studies in which the most commonly investigated knee flexor muscles have been biceps femoris (however not separated into both BFL and BFS) and SemM [3, 5, 10, 11, 23].

Although both heads of the biceps femoris share the same distal tendon and deep aponeurosis, these muscles have different activation timing during the pedaling cycle. BFL activation was most prominent during the downstroke phase at all workloads, while the BFS activation occurred later, crossing the bottom dead center into the upstroke phase. A similar behavior of the SemT suggests that these knee flexors contribute to the power production not only during the downstroke phase as described elsewhere [31] but also during the upstroke phase. In the downstroke phase the BFL presumably contributes to extending the hip whereas BFS activation would resist the required knee extension and may also have a posterior knee stabilizing function. The only knee flexor muscle with significant differences in offset activation timing and crank angle of peak activity between workloads was the SemT between the initial and final workloads.

The SemM was activated largely in synergy with the BFL rather than the BFS, indicating that this biarticular muscle also contributes to hip extension in the downstroke phase assisting the knee extensors in power production and possibly reducing fatigue effects in the knee extensors [31, 32]. During the upstroke phase in which knee flexion is combined with hip flexion, the SemT seems to activate in synergy together with the BFS. However, SemT is activated earlier (around bottom dead center) whereas the deep BFS seems to have a greater delay in the activation timing.

The increases in mean EMG activation level suggest an increased effort required to produce sufficient muscle power at increasing workloads, which is reflected in the increased heart rate and perceived effort. Two participants did not reach the maximal workload of the 20 min incremental protocol, with maximal workloads of 270 W and 310 W and the maximum 20 on the Borg scale respectively. All other participants completed the incremental protocol (350 W), with maximum perceived effort ranging from 17 to 19 on the Borg scale. The high perceived effort at the highest workload indicated considerable fatigue in the final stages of the incremental test. Previous studies have shown that when pedaling at a constant workload the muscles progressively recruit additional motor units and increase the firing rate to maintain muscle power at that level of intensity [4, 25, 33, 34]. The increased EMG activation levels seen in the present study therefore presumably also reflected an increased activation required to compensate for muscle fatigue. According to Enders, von Tscharner [35], the RF plays a key role in coordinating pelvis and thigh segments at a high workload (300 W) and seems to be the first muscle susceptible to muscular fatigue, followed by VL and VM [25]. However, in order to better understand the mechanical role of the RF, the activation patterns of more muscles crossing the hip and knee would be required [25].

The present study support previous studies concerning the superficial thigh muscle EMG activation at higher intensities [4, 22, 25, 26] suggesting that both superficial quadriceps and hamstrings are recruited significantly more at higher workloads. The results concerning the deep Vint indicate that this muscle also contributes to increases in generated power in the downstroke phase.

Fine-wire electrodes provide an attractive solution to evaluating neuromuscular activation of muscles that are difficult to access with surface electrodes. In cycling, Chapman, Vicenzino [16] reported ultrasound guided fine-wire recordings in the lower leg to be feasible and in agreement with activation patterns observed using superficial electrodes. This type of electrode has also been reported to have similar reliability to superficial electrodes [36]. Intramuscular EMG is appropriate in applications requiring large ranges of motion combined with higher velocity as the wire is flexible and follows muscle movement under the skin. There is a risk of displacement of the recording electrode tip during movement, however this risk was minimalized by the external wire sections being carefully taped to the skin with a long loop. The outliers in mean EMG activation levels that were removed in the statistical analysis indicate that there may however have been some disturbance of the internal position of the electrodes in these cases. Finally, care must be taken in electrode insertion to not come in contact with nerves or blood vessels, and some discomfort can be experienced. However, no participants in this study expressed discomfort during the cycling protocol.

The small sample size in the present study needs to be taken into consideration when interpreting the observed effects of increasing workload on EMG activation. Similar previous studies using intramuscular EMG during cycling have also had similar sample sizes (9–12 participants) [1, 16, 21, 37, 38]. However, sample sizes were further reduced in some cases in this study due to difficulty in finding a robust electrode insertion site (BFS) or removed data outliers. This must be taken into account when interpreting the presented results.

In future, EMG information from more muscles combined with kinetic data from the pedals would assist in a more complete understanding of muscle function during cycling. More research using the fine-wire technique with more participants is required to consolidate the results presented here and this is especially relevant for obtaining more information concerning the function of the BFS. Furthermore different cycling protocols could be compared to explore the effects of various combinations of fatigue and workload.

## Conclusion

Intramuscular EMG in eight thigh muscles showed that mean EMG activation level increased with increasing workload for the RF, VM, VL, Vint, BFL SemM and SemT muscles. The deep Vint appeared to be activated in synergy with the superficial knee extensors, whereas BFS showed a different pattern of activation compared to the superficial knee flexors. The BFS also had its angle of peak activity during the downstroke phase at the initial workload however this shifted towards the upstroke phase when the workload was increased. The results of BFS activity during cycling indicated a unique role of this muscle in knee flexion when the hip is simultaneously flexed. This specific role of the BFS should be investigated further. Activation timing increased significantly at higher workloads for RF, VL, VM and SemT.

## Supporting information

S1 Fig Double activation pattern (mean ± SD) at initial, intermediate and final workloads. (TIF)

S1 Table Mean EMG activation level for the whole pedaling cycle at initial, intermediate and final workloads. Outliers deviating from the mean by more than two standard deviation were removed from the statistical analysis. These are indicated by empty cells, the general mean and SD were calculated across all participants.
(PDF)

S2 Table EMG onset and offset at initial, intermediate and final workloads. (PDF)

S3 Table Crank angles at which peak activity occurred at initial, intermediate and final workloads. (PDF)
