# Supplementary material for: Effect of increasing workload on knee extensor and flexor muscular activity during cycling as measured with intramuscular electromyography
Source: PLoS One. 2018 Aug 2;13(8):e0201014. doi: 10.1371/journal.pone.0201014 (PMC6071990; doi:10.1371/journal.pone.0201014)
Supplement: S1 Table — Outliers deviating from the mean by more than two standard deviation were removed from the statistical analysis. These are indicated by empty cells, the general mean and SD were calculated across all participants. (PDF) [file pone.0201014.s002.pdf]

S1 Table. Mean EMG activation level for the whole pedaling cycle at initial, intermediate and final workloads.

**Initial workload**								
	BFS	BFL	SemM	SemT	RF	Vint	VL	VM
Participant 1		6.69	10.89	8.19		14.38	9.19	11.87
Participant 2		7.20	3.85	4.95	3.41	9.28	7.42	7.23
Participant 3		10.89	6.35	13.90	5.48	7.90	10.84	10.40
Participant 4		2.67	8.27	6.28	12.06		4.68	
Participant 5		16.59	8.91	8.29	15.03	21.72		22.02
Participant 6	25.24	7.00	16.43	5.87	10.91	20.79	15.77	16.15
Participant 7	13.85	5.87	14.64	6.72	6.43	27.02	8.73	8.83
Participant 8	14.44	5.84	4.00	6.81	8.66	14.85	13.96	14.93
Participant 9	5.14	8.56		6.32	6.32	16.36	12.82	14.13
**Mean**	**14.67**	**7.92**	**9.17**	**7.48**	**8.54**	**16.54**	**10.42**	**13.20**
**SD**	**8.23**	**3.93**	**4.62**	**2.63**	**3.88**	**6.43**	**3.65**	**4.70**

**Intermediate workload**								
	BFS	BFL	SemM	SemT	RF	Vint	VL	VM
Participant 1		6.75	20.31	15.16		20.76	14.45	17.75
Participant 2		11.84	8.61	9.08	8.38	15.88	11.04	11.15
Participant 3		12.16	7.02	18.32	9.91	16.40	11.63	12.22
Participant 4		8.08	9.13	9.25	19.13		15.42	
Participant 5		14.14	8.50	11.84	20.06	22.88		22.94
Participant 6	15.48	12.52	19.93	8.36	16.46	29.30	19.32	18.42
Participant 7	19.71	9.54	17.53	12.20	6.99	14.25	11.01	10.43
Participant 8	25.00	12.88	13.07	15.50	5.93	12.68	16.86	17.86
Participant 9	9.09	15.78		15.07	9.26	18.76	17.66	18.41
**Mean**	**17.32**	**11.52**	**13.01**	**12.75**	**12.02**	**18.86**	**14.67**	**16.15**
**SD**	**6.73**	**2.89**	**5.51**	**3.46**	**5.64**	**5.39**	**3.20**	**4.39**

**Final workload**								
	BFS	BFL	SemM	SemT	RF	Vint	VL	VM
Participant 1		19.18	19.36	24.57		21.90	15.23	18.17
Participant 2		19.35	24.43	17.33	27.19	28.92	20.38	18.13
Participant 3		17.57	14.56	20.91	18.39	26.23	16.28	16.31
Participant 4		19.02	9.68	11.64	36.01		14.37	
Participant 5		21.40	12.30	16.14	22.06	23.54		27.00
Participant 6	20.87	23.57	23.68	19.66	23.78	27.02	24.36	23.17
Participant 7	29.86	24.71	18.32	21.18	28.91	32.08	23.33	21.16
Participant 8	24.60	18.88	27.11	28.09	13.44	22.55	20.44	22.96
Participant 9	33.34	30.06		27.30	21.25	15.38	21.11	22.09
**Mean**	**27.17**	**21.53**	**18.68**	**20.76**	**23.88**	**24.70**	**19.44**	**21.12**
**SD**	**5.53**	**3.97**	**6.19**	**5.35**	**6.89**	**5.08**	**3.73**	**3.46**
