# Supplementary material for: Effect of increasing workload on knee extensor and flexor muscular activity during cycling as measured with intramuscular electromyography
Source: PLoS One. 2018 Aug 2;13(8):e0201014. doi: 10.1371/journal.pone.0201014 (PMC6071990; doi:10.1371/journal.pone.0201014)
Supplement: S2 Table — (PDF) [file pone.0201014.s003.pdf]

S2 Table. EMG onset and offset at initial, intermediate and final workloads.

Initial workload

	BFS		BFL		SemM		SemT		RF		Vint		VL		VM	
	Onset	Offset	Onset	Offset	Onset	Offset	Onset	Offset	Onset	Offset	Onset	Offset	Onset	Offset	Onset	Offset
Participant 1			83	123	107	191	142	199	19	110	338	98	339	92	356	104
Participant 2			54	138	102	148	84	214	232	334	321	104	26	104	25	103
Participant 3			76	164	91	162	126	194	281	322	314	111	360	90	1	95
Participant 4			77	140	355	146	121	197	25	79	292	83	315	111	323	115
Participant 5			83	142	59	165	103	161			352	110	351	102	334	117
Participant 6	120	265					129	201					334	87	281	320
Participant 7	127	228					106	156					351	111	3	111
Participant 8	129	269					127	198							329	89
Participant 9	119	274													349	106
**Mean**	**124**	**259**	**75**	**141**	**71**	**162**	**117**	**190**	**319**	**31**	**323**	**101**	**348**	**100**	**342**	**129**
**SD**	**5**	**21**	**12**	**15**	**46**	**18**	**18**	**20**	**75**	**74**	**23**	**11**	**22**	**10**	**30**	**72**

Intermediate workload																
	BFS		BFL		SemM		SemT		RF		Vint		VL		VM	
	Onset	Offset	Onset	Offset	Onset	Offset	Onset	Offset	Onset	Offset	Onset	Offset	Onset	Offset	Onset	Offset
Participant 1			83	147	72	211	143	234	5	165	221	115	254	104	238	112
Participant 2			42	145	104	167	89	224	239	339	327	113	12	112	7	114
Participant 3			84	171	96	170	133	206	292	348	358	114	350	84	3	102
Participant 4			68	167	356	154	130	217	351	100	300	98	321	123	18	112
Participant 5			41	155	359	166	85	172			355	106	356	103	333	123
Participant 6	145	234					125	235					330	99	333	127
Participant 7	140	284					110	255					347	104	8	108
Participant 8	137	287					121	215							327	103
Participant 9	158	213													352	104
**Mean**	**145**	**254**	**64**	**157**	**53**	**174**	**117**	**220**	**312**	**58**	**312**	**109**	**333**	**104**	**340**	**112**
**SD**	**9**	**37**	**21**	**12**	**52**	**22**	**21**	**24**	**58**	**90**	**56**	**7**	**36**	**12**	**40**	**8**

Final workload

	BFS		BFL		SemM		SemT		RF		Vint		VL		VM	
	Onset	Offset	Onset	Offset	Onset	Offset	Onset	Offset	Onset	Offset	Onset	Offset	Onset	Offset	Onset	Offset
Participant 1			59	172	95	226	134	238	208	162	338	104	249	107	240	111
Participant 2			8	153	80	195	80	226	240	39	328	117	356	113	356	116
Participant 3			75	179	111	174	141	231	277	34	263	117	343	96	352	104
Participant 4			34	178	354	145	147	266	310	104	298	105	322	118	355	117
Participant 5			41	155	71	212	44	201			310	91	342	114	334	128
Participant 6	149	254					139	259					335	105	332	124
Participant 7	163	295					111	267					346	110	345	115
Participant 8	143	293					116	291							329	106
Participant 9	136	333													346	110
**Mean**	**148**	**294**	**43**	**167**	**70**	**190**	**114**	**247**	**259**	**85**	**307**	**107**	**328**	**109**	**332**	**115**
**SD**	**11**	**32**	**25**	**13**	**45**	**32**	**36**	**29**	**44**	**61**	**29**	**11**	**36**	**7**	**36**	**8**
