# Supplementary material for: Effect of increasing workload on knee extensor and flexor muscular activity during cycling as measured with intramuscular electromyography
Source: PLoS One. 2018 Aug 2;13(8):e0201014. doi: 10.1371/journal.pone.0201014 (PMC6071990; doi:10.1371/journal.pone.0201014)
Supplement: S3 Table — (PDF) [file pone.0201014.s004.pdf]

S3 Table. Crank angles at which peak activity occurred at initial, intermediate and final workloads.

**Initial workload**								
	BFS	BFL	SemM	SemT	RF	Vint	VL	VM
Participant 1		85	136	127	61	45	58	45
Participant 2		117	125	152	294	270	50	42
Participant 3		109	111	141	287	278	45	34
Participant 4		106	120	158	50	39	45	50
Participant 5		239	124	138	296	343	23	69
Participant 6	140	80	111	79	264	243	343	7
Participant 7	162	75	120	133	289	52	34	60
Participant 8	134	112	109	128	311	311	2	69
Participant 9	56	75	296	134	331	66	50	43
**Mean**	**123**	**111**	**139**	**132**	**323**	**343**	**32**	**47**
**SD**	**46**	**51**	**59**	**22**	**56**	**70**	**25**	**19**

Intermediate workload								
	BFS	BFL	SemM	SemT	RF	Vint	VL	VM
Participant 1		117	134	107	278	44	46	53
Participant 2		102	68	180	299	292	52	51
Participant 3		112	114	189	24	277	50	37
Participant 4		77	118	154	72	68	72	147
Participant 5		112	129	250	300	67	45	33
Participant 6	162	118	11	131	273	3	7	104
Participant 7	206	62	122	152	275	37	42	62
Participant 8	228	113	121	136	312	314	55	5
Participant 9	197	128	301	176	61	54	21	60
**Mean**	**198**	**105**	**124**	**164**	**330**	**8**	**43**	**61**
**SD**	**27**	**21**	**77**	**42**	**64**	**60**	**19**	**42**

Final workload								
	BFS	BFL	SemM	SemT	RF	Vint	VL	VM
Participant 1		103	147	132	18	67	45	35
Participant 2		103	62	183	289	48	74	35
Participant 3		118	111	147	27	26	25	16
Participant 4		65	73	165	33	48	275	310
Participant 5		99	140	199	302	12	7	66
Participant 6	212	90	3	97	285	30	13	12
Participant 7	210	108	150	162	319	25	12	60
Participant 8	252	118	117	149	318	320	11	67
Participant 9	221	167	129	196	345	342	9	74
**Mean**	**224**	**108**	**104**	**159**	**335**	**22**	**12**	**35**
**SD**	**19**	**27**	**49**	**32**	**42**	**34**	**43**	**39**
